# Sex Differences in Elderly People’s Sleep: A Cross-Sectional Study

**DOI:** 10.3390/medicina60101654

**Published:** 2024-10-09

**Authors:** Francesco Salis, Maristella Belfiori, Michela Figorilli, Martina Mulas, Monica Puligheddu, Antonella Mandas

**Affiliations:** 1 Department of Medical Sciences and Public Health, University of Cagliari, 09042 Monserrato, Italy; 2 Department of Biomedical Sciences, University of Cagliari, 09042 Monserrato, Italy; 3 University Hospital “Azienda Ospedaliero-Universitaria” of Cagliari, 09042 Monserrato, Italy; 4 Sleep Disorder Centre, Department of Medical Sciences and Public Health, University of Cagliari, 09042 Monserrato, Italy

**Keywords:** cognitive impairment, depression, elderly, geriatrics, sleep quality, sex differences

## Abstract

*Background and Objectives*: Sex differences are unclear in geriatric sleep medicine, and most evidence comes from inference from preclinical bases or clinical studies conducted on younger people. The aim of this study is to explore sex differences in sleep quality and daytime sleepiness in a cohort of elderly people. *Materials and Methods*: This cross-sectional study involved subjects aged 65 years or older undergoing multidimensional evaluation, including sleep quality and daytime sleepiness assessment with validated tools. *Results*: This study included 226 subjects (69.5% women), the majority of whom showed poor sleep quality (64.2%). A logistic regression model put one before the other sleep quality and gender. It initially showed that men were about half likely as women to have poor sleep quality (OR 0.48, 95%CI 0.27–0.86). Nonetheless, after adjusting for cognitive status and mood, the difference became smaller and insignificant (OR 0.72, 95%CI 0.38–1.38). *Conclusions*: Sex differences in elderly people’s sleep quality seem to not be independent, appearing to be affected by alterations in cognitive status and mood.

## 1. Introduction

As people age, their sleep habits change, and these changes can differ between men and women. Understanding these distinctions is critical for establishing tailored interventions in older persons. Nonetheless, most of the studies in sleep medicine are not focused on elderly subjects, nor on gender differences. Starting from animals, female mice generally sleep less than their male counterparts [1], probably due to hormonal differences, especially steroids [1,2]. Nonetheless, little evidence has been produced in animal models to clearly explain the origin of sex differences in sleep [3]. Experiments in *Drosophila* demonstrated that female flies show a progressive decrease in daytime sleep when aging [4]. Despite the scientific interest of these results, human sleep appears to be extremely more complex and characterized by proper behaviors and syndromes. In humans, it is known that sleep-stage transitions are influenced by both age and sex [5]. According to a study on young people, men seem to have a shorter sleep time and a worse sleep efficiency than women [6]. Also, in the general population, women have longer sleep latency [7], and a recently published study, conducted on middle-aged people, showed that long sleep duration is related to higher blood pressure in women but not in men [8]. The most common sleep disorders are in turn more common in women: among them, insomnia, restless leg syndrome, and rapid eye movement (REM) behavior disorders [3]. The obstructive sleep apnea syndrome is instead more common in men, and women are also historically less likely to refer to specialized centers [9]. Focusing on elderly subjects, older women seem to have slightly shorter sleep than men [7], and lower delta activity [10], suggesting a shorter deep sleep, resulting in poorer daytime performances and quality of life [11]. Coherently, nearly half of postmenopausal women complain about insomnia [3], and many factors can supposedly be linked to the above, suggesting a multidimensional approach [12]. For example, women usually take care of something else more frequently than men, thus reporting more distress [3]. Also, women show a higher incidence of depression, especially in the elderly [13], and it is known to be associated with poor sleep quality, together with neurocognitive disorders, which in turn are linked with mood disorders [14,15]. The above mentioned conditions have been widely described in the literature as determinants of sleep quality, and vice versa. A meta-analysis including more than 25,000 showed that short and long sleep time can lead to a higher risk of depression [16], and sleep alterations are considered a determinant of depression pathophysiology [17]. Also, antidepressants are recognized as modifiers of sleep architecture [18]. Moreover, sleep quality has been called upon as the link between depression and cognitive impairment [19], as supposed by a Chinese study on a large cohort of people aged 50 years or more. The assessment of cognitive capacities has been demonstrated as crucial in different settings, from emergency departments to specialistic services [20,21], and it has been proved that treating sleep diseases can also slow down cognitive impairment [22]. In this context, sex differences have usually been on the framework, often simply considered as one of the countless covariates included in the analysis. Currently, there is a growing area of research in the scientific literature, aimed at exploring this issue more thoroughly. Specifically, it is suggested that understanding the mechanisms behind race- and sex-related differences in sleep could enhance the personalization of the care [23]. Additionally, identifying clear sex differences in daytime sleepiness may provide insights into the natural progression of diseases [24], and contribute to the overall management of geriatric syndromes [25].

To sum up, determining differences between older men and women would help physicians in offering a more complete patient framing and possible further personalization of the therapy. The current scientific literature has not yet specifically explored enough sex differences in elderly people’s sleep quality, as most studies focused on surrogate measures such as hours of sleep or differences in sleep phases.

The aim of this study is to explore sex differences in sleep quality and daytime sleepiness in a cohort of elderly people.

## 2. Participants and Methods

### 2.1. Design of This Study

A cross-sectional study was conducted at the Geriatric Service of the University Hospital of Monserrato, Cagliari, Italy, in collaboration with the Sleep Centre of the same University Hospital, between June 2021 and November 2022.

### 2.2. Inclusion/Exclusion Criteria

Patients aged 65 years or more were included. People who did not complete the geriatric assessment including the sleep evaluation for any cause, with missing data, or who did not give informed consent were excluded. People with severe cognitive impairment, defined as Mini-Mental State Examination (MMSE) score < 14 [26], were also excluded, since they were considered unable to fill in the purposed questionnaires. No other restrictions based on specific diseases or conditions were placed.

### 2.3. Sampling

Considering confidence level: 95%, Confidence Interval: 5%, anticipated prevalence (P): 0.5, Z-score (z): 1.96, and error margin (e): 7%, the final sample (N) should consist of at least 196 subjects, according to the following formula [27]:

$$N=\frac{z^{2}\times P\;(1-P)}{e^{2}}$$

### 2.4. Assessment

The enrolled subjects were evaluated by medical doctors with specific training in geriatrics for

- Pittsburgh Sleep Quality Index (PSQI) [28] to evaluate subjective sleep quality. Scores >5 indicate poor sleep quality [28].
- Epworth Sleepiness Scale (ESS) to evaluate daytime sleepiness. Scores ≥11 indicate EDS [29,30].
- MMSE [23] to evaluate cognitive abilities. Scores ≥26 are suggestive for absence of cognitive impairment [31].
- Geriatric Depression Scale (GDS) [32]—15 items to evaluate depressive symptoms in elderly people. Scores ≤ 5 indicate absence of depression [32].
- Activities of Daily Living–Barthel Index (ADL) [33] to evaluate the ability to perform everyday personal tasks. Scores ≥ 90 suggest independence [33,34],
- Instrumental Activities of Daily Living (IADL) [35] to evaluate the ability to perform more complex and instrumental everyday tasks. Scores ≥6 suggest independence [34,35].
- Cumulative Illness Rating Scale (CIRS) [36] to evaluate the elderly state of health, comorbidities, and drugs taken.

### 2.5. Statistical Analysis

The Shapiro–Wilk test was used to assess the normal distribution of the variables. The variables are expressed as medians and Interquartile Ranges (IQRs) or in percentages (%), where appropriate. The variable “age” was normally distributed; hence, it is expressed as the mean and Standard Deviation (SD). Categorial variables were compared with the chi-squared (χ^2^) test. Continuous variables were compared with the Wilcoxon rank sum test.

In order to group the sample, we considered PSQI (“poor sleep quality” for scores > 5 and “good sleep quality” for scores ≤ 5), ESS (“normal daytime sleepiness” for scores < 11 and EDS for scores ≥ 11), MMSE (“absence of cognitive impairment” for scores ≥ 26 and “cognitive impairment” for scores < 26), and GDS (“absence of depression” for scores ≤ 5 and “depression” for scores > 5).

Sleep quality, used as the dependent variable for the logistic regression models, was considered according to the abovementioned dichotomization, and the reference category for gender was “female”. Their results are expressed as Odds Ratios (ORs) and 95% Confidence Intervals (95%CIs). The control of confounding effects was studied with a directed acyclic graph (DAG), designed according to previous research on the topic, and we considered cognitive status and mood [19]. Five percent was the threshold beneath which the frequency of sleep-modifying drugs taken were not taken into account in the analysis. The goodness of fit of the regression models is expressed with the Area Under the ROC Curve (AUC) [37]. The results are reported indicating *p*-values in reference to 95% Confidence Intervals (95%CIs).

RStudio software (version 1-494) was used for the statistical analysis.

## 3. Results

According to inclusion/exclusion criteria, the final sample was made up of 226 subjects, of whom 157 (69.5%) were females, with a mean age of 80.8 years (SD: 5.9). The characteristics of the enrolled subjects are shown in Table 1.

A sum of 145 people out of 226 (64.2%) achieved PSQI scores > 5, indicating poor sleep quality, and 42 out of 226 (18.6%) achieved ESS scores ≥ 11, indicating the presence of EDS. Also, according to the abovementioned thresholds, 155 people (68.6%) achieved MMSE scores < 26, indicating cognitive impairment, and 116 (51.3%) had GDS scores > 5, indicating depressed mood. Among drugs with an influence on sleep, taken by at least 5% of the population, beta-blockers (32.3%), benzodiazepines (28.3%), and antidepressants (19.5%, of whom 65.9% are SSRIs) were the most common in the study population.

A sum of 110 people were found to have poor sleep quality, according to PSQI, regardless of the absence of EDS, while 74 people had a good sleep quality together with an absence of EDS (χ^2^: 7.25, *p* = 0.0071). Among people with bad sleep quality, 90 also revealed depressed mood, while the other 55 showed acceptable mood (χ^2^: 17.50, *p* < 0.001). On the contrary, EDS did not show an association with depression: among people with EDS, 22 were depressed, and 20 were not (*p* = 1).

We noticed that the mentioned data had a differential expression according to gender. In particular, women complained significantly more than men about bad sleep quality (69.4% vs. 52.2%, respectively, χ^2^: 5.47, *p* = 0.0193), and were also more frequently depressed (57.3% vs. 37.7%, respectively, χ^2^: 6.64, *p* = 0.0099); moreover, for the sake of completeness, only 3 men (4.3%) achieved GDS scores >9, suggestive for moderate/severe depression, versus 38 women (24.2%). The assumption of antidepressant drugs was coherently more common in women (24.2% vs. 8.7%, respectively, χ^2^: 6.39, *p* = 0.0114). Nonetheless, in our sample, the assumption of antidepressants was not related to sleep quality (*p* = 0.1347), nor to daytime sleepiness (*p* = 1). About the latter, women complained less than men about EDS (χ^2^: 4.44, *p* = 0.0350). For the sake of completeness, 23 women (14.6%) and 19 men (27.5%) reported EDS according to ESS scores. Significant gender differences in cognitive impairment did not emerge (*p* = 0.1334), while a relationship between cognitive status and overall sleep quality was found among people with bad sleep. A total of 107 people showed cognitive impairment, and 38 did not (χ^2^: 4.44, *p* = 0.0305). As discussed for depression, cognitive-related differences in EDS did not come up (*p* = 0.1735).

**Figure 1:**
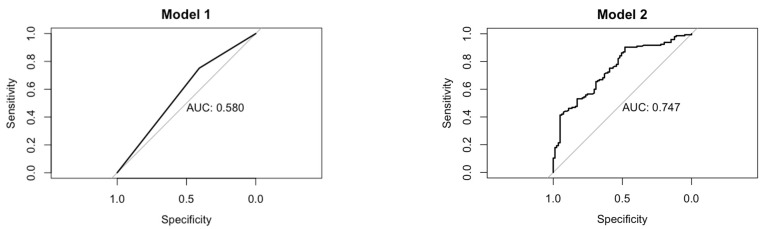
Goodness of fit of logistic models. AUC, Area Under the ROC Curve; Model 1, unadjusted model; dependent variable: Pittsburgh Sleep Quality Index; independent variable: gender; Model 2, adjusted model for Mini-Mental State Examination and Geriatric Depression Scale.

To pursue the aim of this study, we designed a logistic regression, considering dichotomized PSQI (model 1) as a dependent variable, and gender as an independent variable (Table 2, Figure 1). The model (AUC: 0.580) showed that men have 52% less “odds” of experiencing poor sleep quality than women (OR: 0.48, 95%CI 0.27–0.86). In order to correct the model for potential confounders, we designed the appropriate DAG, by which it was found that MMSE and GDS should have been considered as potential confounders. We performed, therefore, the adjusted regression (model 1a, AUC: 0.747), which returned OR: 0.72 (95%CI: 0.38–1.38), meaning that, when considering cognitive status and depression, the gender-dependent difference in sleep quality smooths out and become nonsignificant (Table 2, Figure 1).

## 4. Discussion

Gender differences in sleep medicine are known, though underlining relatively unclear mechanisms [3]. The aim of this paper was to describe sex differences in sleep quality and daytime sleepiness in a cohort of elderly people. Also, we aimed to explore the influence of possible other variables coming from the scientific literature, to offer a result that is, as much as possible, free of the influence of possible confounders. Our analysis actually showed an impact of sex on sleep quality, yet probably mediated by other issues such as mood and cognitive impairment.

In detail, in order to pursue our aim, we recruited a sample of 226 subjects aged 65 years or older, the majority of whom were women (69.5%), complaining of poor sleep quality (64.2%). We first found an association between poor sleep quality, assessed with PSQI, and depressed mood, assessed with GDS (*p* < 0.001), consistently with the literature on the topic [3,14]. This association also showed a sex difference, since women were found to be significantly more frequently depressed (*p* = 0.0099) and with poor sleep quality (*p* = 0.0193) than men, and the assumption of antidepressants logically followed the same trend, although the assumption of these medications was unrelated to sleep quality and daytime sleepiness. Some previous studies showed that the association of depression and poor sleep quality can result as an alteration of sleep architecture [38]. As for antidepressants, we did not find significant associations between their assumption and sleep quality: the literature offered some studies pointing out possible effects of different pharmacological classes [38], but without a clear consensus [38,39]. Thus, our result does not sound surprising, all the more so because it is known that antidepressants can slightly differ in efficacy and safety between younger and older people [40].

The logistic univariable model characterized by gender as an independent variable and sleep quality as a dependent variable suggested that men would be more likely to have better sleep quality than women, but, after the correction for confounders, this relationship becomes nonsignificant (OR: 0.72, 95%CI: 0.38–1.38, AUC: 0.747). What has been found is of particular interest, since it shows that a clear epidemiological association, which is confirmed in different age groups, in the geriatric population can be significantly influenced by factors extrinsic to “sleep itself”, such as cognitive abilities and mood disorders, which are common in the elderly [12]. All the above can be explained by the significant complexity and comorbidity burden of elderly age [12], which does not allow us to consider it as a mere “old adult age”. In this perspective, even other conditions in which sex differences have been displayed could be reviewed in a more “holistic” way.

Our data, meanwhile, showed that excessive daytime sleepiness was less common in women, consistently with preclinical studies [4], while inconsistently with data coming from clinical studies—more focused on daytime sleepiness as a consequence of other diseases, such as obstructive sleep apnea syndrome, which is actually more common in men [9,41,42,43]. In any event, depression and cognitive impairment appeared to be unrelated to excessive daytime sleepiness, according to our results. Previous studies focused on the possible relationship between sleep, mood, and cognition. Nonetheless, it is not completely defined whether mood disorders and cognitive impairment influence sleep or whether poor sleep quality worsens mood and cognitive performances. A recent study hypothesized that sleep is the mediator of the other two conditions [19]. Our results, obtained with a different study design, seem to be close to this line of research, according to which worse cognitive performances and depressed mood influence sleep quality [16,17,19].

Our study has several strengths. Firstly, it examines data coming from a relatively large group of patients with an average age of over 80 years, which is quite uncommon in the sleep literature. Also, our results show a clinical practical implication. In detail, it shows that apparently sex-related modifications in sleep are possibly mediated by other factors, such as mood and cognition. Hence, a comprehensive assessment should also be provided in sleep geriatric medicine.

We also recognize some limitations. Firstly, this is a monocentric study, so we are not able to generalize our results to the entire geriatric population. Also, we did not perform an objective assessment of the sleep (such as polysomnography), which would have added scientific soundness to our research. Also, we did not follow up with the patients in order to establish the impact of the therapies on the sleep.

## 5. Conclusions

In conclusion, our study demonstrates that, even if sex can appear to be related to sleep quality in elderly people, their association is not independent, but it seems to be affected by other alterations, i.e., in cognitive status and mood. Hence, with the aim of an increasingly personalized approach, sleep medicine has to allow for different clinical phenotypes in geriatric domains in men and women, and to consider sleep disorders as geriatric multifactorial syndromes.

## Figures and Tables

**Table 1:** Characteristics of the sample.

Variable	Mean	SD
Age (years)	80.8	5.92
	Median	IQR
MMSE	23.9	20.4–26.6
GDS	6	3–9
ADL	88	75–97
IADL	4	2–6
CIRS	30	26–32
Comorbidities (n.)	9	7–13
	n.	%
PSQI > 5 (Bad sleep quality)	145	64.2
ESS ≥ 11 (Excessive daytime sleepiness)	42	18.6
MMSE < 26 (Cognitive impairment)	155	68.6
GDS ≥ 5 (Depressed mood)	116	51.3
Hypertension	160	70.8
Chronic cerebral vasculopathy	71	31.4
Atrial fibrillation	32	14.2
Other heart diseases	88	38.9
Diabetes mellitus	66	29.2
Hypercholesterolemia	119	52.7
Osteoarthritis	148	65.5
Beta-Blockers	73	32.3
Antidepressants (including SSRI and SNRI)	44	19.5
SSRI	29	12.8
SNRI	12	5.3
Antipsychotics	12	5.3
Benzodiazepines	64	28.3
Opioids	14	6.2

ADL, Activities of Daily Living–Barthel Index; CIRS, Cumulative Illness Rating Scale; ESS, Epworth Sleepiness Scale; GDS, Geriatric Depression Scale; IADL, Instrumental Activities of Daily Living; IQR, Interquartile Range; MMSE, Mini-Mental State Examination; PSQI, Pittsburgh Sleep Quality Index; SD, Standard Deviation; SNRI, Serotonin-Norepinephrine Reuptake Inhibitors; SSRI, Selective Serotonin Reuptake Inhibitors.

**Table 2:** Regression models.

Model	Dependent Variable	Independent Variables	OR	95%CI
1	PSQI	(Intercept)	2.27	1.63–3.22
		Gender (male)	0.48	0.27–0.86
2 (corrected)	PSQI	(Intercept)	0.96	0.15–6.13
		Gender (male)	0.72	0.38–1.38
		MMSE	0.97	0.89–1.04
		GDS	1.31	1.19–1.46

95%CI, 95% Confidence Interval; GDS, Geriatric Depression Scale; MMSE, Mini-Mental State Examination; OR, Odds Ratio; PSQI, Pittsburgh Sleep Quality Index.

## Data Availability

The data and materials used and/or analyzed during the current study are not publicly available. They are available from the corresponding author upon reasonable request.

## Abbreviations

ADL, Activities of Daily Living–Barthel Index; AUC, Area Under the ROC Curve; CI, Confidence Interval; CIRS, Cumulative Illness Rating Scale; EDS, excessive daytime sleepiness; ESS, Epworth Sleepiness Scale; GDS, Geriatric Depression Scale; IADL, Instrumental Activities of Daily Living; IQR, Interquartile Range; MMSE, Mini-Mental State Examination; OR, Odds Ratio; PSQI, Pittsburgh Sleep Quality Index; SD, Standard Deviation; SNRI, Serotonin–Norepinephrine Reuptake Inhibitor; SSRI, Selective Serotonin Reuptake Inhibitor.

## References

[1] Paul K.N., Dugovic C., Turek F.W., Laposky A.D. (2006). Diurnal sex differences in the sleep-wake cycle of mice are dependent on gonadal function. Sleep.

[2] Cusmano D.M., Hadjimarkou M.M., Mong J.A. (2014). Gonadal steroid modulation of sleep and wakefulness in male and female rats is sexually differentiated and neonatally organized by steroid exposure. Endocrinology.

[3] Mallampalli M.P., Carter C.L. (2014). Exploring sex and gender differences in sleep health: A Society for Women’s Health Research Report. J. Womens Health.

[4] Koh K., Evans J.M., Hendricks J.C., Sehgal A. (2006). A Drosophila model for age-associated changes in sleep:wake cycles. Proc. Natl. Acad. Sci. USA.

[5] Wächter M., Kantelhardt J.W., Bonsignore M.R., Bouloukaki I., Escourrou P., Fietze I., Grote L., Korzybski D., Lombardi C., Marrone O. (2020). Unique sleep-stage transitions determined by obstructive sleep apnea severity, age and gender. J. Sleep Res..

[6] Goel N., Kim H., Lao R.P. (2005). Gender differences in polysomnographic sleep in young healthy sleepers. Chronobiol. Int..

[7] Ohayon M.M., Reynolds C.F., Dauvilliers Y. (2013). Excessive sleep duration and quality of life. Ann. Neurol..

[8] Guo J., Fei Y., Li J., Zhang L., Luo Q., Chen G. (2016). Gender- and age-specific associations between sleep duration and prevalent hypertension in middle-aged and elderly Chinese: A cross-sectional study from CHARLS 2011-2012. BMJ Open.

[9] Redline S., Kump K., Tishler P.V., Browner I., Ferrette V. (1994). Gender differences in sleep disordered breathing in a community-based sample. Am. J. Respir. Crit. Care Med..

[10] Latta F., Leproult R., Tasali E., Hofmann E., Van Cauter E. (2005). Sex differences in delta and alpha EEG activities in healthy older adults. Sleep.

[11] Goldman S.E., Stone K.L., Ancoli-Israel S., Blackwell T., Ewing S.K., Boudreau R., Cauley J.A., Hall M., Matthews K.A., Newman A.B. (2007). Poor sleep is associated with poorer physical performance and greater functional limitations in older women. Sleep.

[12] Salis F., Loddo S., Zanda F., Peralta M.M., Serchisu L., Mandas A. (2022). Comprehensive Geriatric Assessment: Application and correlations in a real-life cross-sectional study. Front. Med..

[13] Alexopoulos G.S. (2005). Depression in the elderly. Lancet.

[14] Shi L., Chen S.J., Ma M.Y., Bao Y.P., Han Y., Wang Y.M., Shi J., Vitiello M.V., Lu L. (2018). Sleep disturbances increase the risk of dementia: A systematic review and meta-analysis. Sleep Med. Rev..

[15] Belfiori M., Salis F., Demelas G., Mandas A. (2024). Association between Depressive Mood, Antidepressant Therapy and Neuropsychological Performances: Results from a Cross-Sectional Study on Elderly Patients. Brain Sci..

[16] Zhai L., Zhang H., Zhang D. (2015). Sleep Duration and Depression among Adults: A Meta-Analysis of Prospective Studies. Depress. Anxiety.

[17] Tsuno N., Besset A., Ritchie K. (2005). Sleep and depression. J. Clin. Psychiatry.

[18] Winokur A., Gary K.A., Rodner S., Rae-Red C., Fernando A.T., Szuba M.P. (2001). Depression, sleep physiology, and antidepressant drugs. Depress. Anxiety.

[19] Liu X., Xia X., Hu F., Hao Q., Hou L., Sun X., Zhang G., Yue J., Dong B. (2022). The mediation role of sleep quality in the relationship between cognitive decline and depression. BMC Geriatr..

[20] Salis F., Pili D., Collu M., Serchisu L., Laconi R., Mandas A. (2023). Six-item cognitive impairment test (6-CIT)‘s accuracy as a cognitive screening tool: Best cut-off levels in emergency department setting. Front. Med..

[21] Grazia A., Altomare D., Preis L., Monsch A.U., Cappa S.F., Gauthier S., Frölich L., Winblad B., Welsh-Bohmer K.A., Teipel S.J. (2023). Feasibility of a standard cognitive assessment in European academic memory clinics. Alzheimer’s Dement..

[22] Lucey B.P., Wisch J., Boerwinkle A.H., Landsness E.C., Toedebusch C.D., McLeland J.S., Butt O.H., Hassenstab J., Morris J.C., Ances B.M. (2021). Sleep and longitudinal cognitive performance in preclinical and early symptomatic Alzheimer’s disease. Brain.

[23] Tapia A.L., Yu L., Lim A., Barnes L.L., Hall M.H., Butters M.A., Buysse D.J., Wallace M.L. (2023). Race and sex differences in the longitudinal changes in multidimensional self-reported sleep health characteristics in aging older adults. Sleep Health.

[24] Snyder B., Cunningham R.L. (2018). Sex differences in sleep apnea and comorbid neurodegenerative diseases. Steroids.

[25] Catikkas N.M., Tunc M., Soysal P. (2023). The prevalence of excessive daytime sleepiness and associated factors in older diabetic patients. Aging Clin. Exp. Res..

[26] Folstein M.F., Folstein S.E., McHugh P.R. (1975). “Mini-mental state”. A practical method for grading the cognitive state of patients for the clinician. J. Psychiatr. Res..

[27] Cochran W.G. (1997). Sampling Techniques.

[28] Buysse D.J., Reynolds C.F., Monk T.H., Berman S.R., Kupfer D.J. (1989). The Pittsburgh Sleep Quality Index: A new instrument for psychiatric practice and research. Psychiatry Res..

[29] Johns M.W. (1991). A new method for measuring daytime sleepiness: The Epworth sleepiness scale. Sleep.

[30] Walker N.A., Sunderram J., Zhang P., Lu S.E., Scharf M.T. (2020). Clinical utility of the Epworth sleepiness scale. Sleep Breath.

[31] Salis F., Costaggiu D., Mandas A. (2023). Mini-Mental State Examination: Optimal Cut-Off Levels for Mild and Severe Cognitive Impairment. Geriatrics.

[32] Sheikh J.I., Yesavage J.A. (1986). Geriatric depression scale (GDS): Recent evidence and development of a shorter version. Clin. Gerontol. J. Aging Ment. Health.

[33] Mahoney F.I., Barthel D.W. (1965). Functional Evaluation: The Barthel Index. Md State Med. J..

[34] Pashmdarfard M., Azad A. (2020). Assessment tools to evaluate Activities of Daily Living (ADL) and Instrumental Activities of Daily Living (IADL) in older adults: A systematic review. Med. J. Islam. Repub. Iran.

[35] Lawton M.P., Brody E.M. (1969). Assessment of older people: Self-maintaining and instrumental activities of daily living. Gerontologist.

[36] Parmelee P.A., Thuras P.D., Katz I.R., Lawton M.P. (1995). Validation of the Cumulative Illness Rating Scale in a geriatric residential population. J. Am. Geriatr. Soc..

[37] Harrell F.E. (2015). Regression Modeling Strategies.

[38] Pandi-Perumal S.R., Monti J.M., Burman D., Karthikeyan R., BaHammam A.S., Spence D.W., Brown G.M., Narashimhan M. (2020). Clarifying the role of sleep in depression: A narrative review. Psychiatry Res..

[39] Bielski R.J., Friedel R.O. (1977). Subtypes of depression--diagnosis and medical management. West J. Med..

[40] Mottram P., Wilson K., Strobl J. (2006). Antidepressants for depressed elderly. Cochrane Database Syst. Rev..

[41] Honig E., Green A., Dagan Y. (2021). Gender differences in the sleep variables contributing to excessive daytime sleepiness among patients with obstructive sleep apnea. Sleep Breath.

[42] Packard A., Bautista R., Smotherman C., Gautham S. (2021). Gender differences in Epworth Sleepiness Scale revealed by paired patient-spouse scoring. Epilepsy Behav..

[43] Fatani A., Al-Rouqi K., Al Towairky J., Ahmed A.E., Al-Jahdali S., Ali Y., Al-Shimemeri A., Al-Harbi A., Baharoon S., Khan M. (2015). Effect of age and gender in the prevalence of excessive daytime sleepiness among a sample of the Saudi population. J. Epidemiol. Glob. Health.

